# Hypoallergenic diet may control refractory epilepsy in allergic children: A quasi experimental study

**DOI:** 10.1038/s41598-019-43252-0

**Published:** 2019-05-03

**Authors:** H. Gorjipour, S. Darougar, M. Mansouri, P. Karimzadeh, M. Hassanvand Amouzadeh, M. R. Sohrabi

**Affiliations:** 1 Department of Immunology and Allergy, Mofid Children Hospital, Shahid Beheshti University of Medical Sciences, Tehran, Iran; 2 Department of Pediatrics, Tehran Medical Sciences Branch, Islamic Azad University, Tehran, Iran; 3 Department of Pediatric Neurology, Shahid Beheshti University of Medical Sciences, Tehran, Iran; 4 Neurology and Neuroscience Research Center, Qom University of Medical Sciences, Qom, Iran; 5 Social Determinants of Health Research Center, Community Medicine Department, School of Medicine, Shahid Beheshti University of Medical Sciences, Tehran, Iran

**Keywords:** Immunology, Epilepsy

## Abstract

Recent data has suggested a definitive role for inflammatory processes in the pathophysiology of epilepsy. In this study we hypothesized that food allergies, as chronic inflammatory processes, underlie the pathophysiology of refractory idiopathic epilepsy and investigated whether food elimination diets may assist in managing refractory epilepsy. The study was conducted on 34 patients up to 16 years of age with refractory convulsions who attended the Allergy Outpatient Clinic, Mofid Children Hospital between 2015 and 2016 with youngest and oldest participants at ages of 3 months and 16 years old, respectively. The participants were categorized into three groups according to the results of skin prick test and serum specific IgE measurements. Elimination diets were instituted for the patients with non IgE-mediated and mixed food allergies. The study was conducted for a period of 12 weeks. The participants were assessed for at least 50% reduction in number of seizures following the intervention. There was a significant reduction in number of seizures (p < 0.001) following the intervention. Seventeen patients (50%) did not experience any seizures after 8 weeks of treatment and 12 patients (35%) had a significant (51–99%) decrease in the number of their seizures. Five patients did not show any changes in their daily seizure frequency. The obtained data suggest that food allergy may play a role in triggering refractory epilepsies and their adequate response to treatment. A trial of elimination diet showed more than 50% seizure reduction in more than 85% of the children studied. However, we believe these results are preliminary and they motivate a fully controlled study in the future.

## Introduction

Food allergy is a serious health problem affecting up to 8% of children and increasingly capturing the attention as a modern-day epidemic with poorly understood drivers^1^. In more than 90% of cases, food allergy is due to the 6 main foods including cow’s milk, egg, soy, wheat, peanut, tree nuts and fish^2^. In contrast to IgE-mediated food allergies for which an accurate diagnosis can be reached by various tests and history, when history and physical examination is indicative of cell-mediated or mixed mechanisms, a trial of elimination diet based on the history is recommended^3–5^.

Idiopathic refractory epilepsies are estimated to comprise 10% to 20% of childhood epilepsies^6^, which may adversely affect brain development^6^. Refractory epilepsy is defined as convulsions that are not controlled with 3 or 4 appropriate first line drugs despite adequate duration and dosage^7^. There is accumulating evidence that inflammatory processes contribute to pathophysiology of refractory seizures^6^. Many previous studies have indicated that allergic reactions are major causes of inflammatory processes in epileptic children^7^. The idea of the role of food allergy in inducing epilepsy is referred to Ward and Patterson in 1927, who performed skin prick test for food allergens on 1000 epileptic individuals and 100 healthy controls and found that 37–67% of patients revealed wheal and flare reactions indicative of prior sensitivity while only 8% of the healthy control group showed positive reactions^8^. In 1989, Egger reported that of the 45 epileptic children, 25 ceased to have seizures and 11 had fewer seizures during oligo-antigenic diet therapy. In 2001, Frediani *et al*. evaluated 72 epileptic children and 202 healthy control, age-matched individuals with their families. They found higher rates of cow’s milk allergy and asthma in epileptic children and dermatitis and rhinitis in their mothers and their siblings, respectively^9^. In 2008, Durkin *et al*. evaluated 91642 children and found higher rate of epilepsy among children with allergic disorders compared with non-allergic children^10^ and concluded that allergic inflammation may contribute to epileptogenesis. However, many authors have refuted this hypothesis^11^.

Recent research data shows a correlation between peripheral inflammation and disease development in central nervous system as the underlying mechanism^12–14^. Thinking of food allergy as a seizure trigger is rather a novel idea and confirmation of this relationship may be difficult. However, once confirmed it may break a new ground in treatment of refractory convulsions in paediatric age group.

In this study, we hypothesized that inflammatory process (involving gut) is an underlying cause in some cases of refractory epilepsy^13–16^ and investigated whether a significant relationship exists between these intractable seizures and food allergy.

## Patients and Methods

### Participants

This prospective, self-controlled and quasi-experimental study was conducted from January 2015 to February 2016 at the Allergic Outpatient Clinic, Mofid Children Hospital, Shahid Beheshti University of Medical Sciences, Tehran. Shahid Beheshti University is responsible for overseeing the health services of more than 5.4 million people in the province of Tehran, including the northern, western and eastern parts of Tehran. Mofid Children Hospital is the only tertiary center that covers all the above areas for treatment of critically ill patients specially patients with refractory seizures.

In this study refractory seizure was defined according to definition of “drug resistant epilepsy” as “failure of adequate trials of two tolerated, appropriately chosen and used antiepileptic drug schedules (whether as monotherapies or in combination) to achieve sustained seizure free”^17^.

#### Inclusion criteria

Patients with a definition of an idiopathic refractory seizure according to the above mentioned diagnostic criteria, aged 3 months to 16 years with a diagnosis of non-IgE mediated food allergy were included.

#### Exclusion criteria

Patients with IgE-mediated food allergy including patients with anaphylaxis (due to their strict self-restrictive diets for prevention of severe hypersensitivity reactions), non-adherence to the avoidance diets, any growth faltering due to negative impacts of elimination diets and refractory seizures due to secondary causes (cerebral palsy, metabolic disorders, etc.) were excluded.

### Sample size calculation

The sample size of this study was determined according to similar studies performed previously evaluating the efficacy of ketogenic diets on refractory seizures^18–22^ due to the lack of previous studies with hypoallergenic regimens.

Those studies demonstrated 50% seizure reduction in 38–50% of the patients after establishment of ketogenic diet. Considering type one error of 5% and type 2 error of 20%, the calculated sample size was 16 patients but due to probable loss of patients during the follow-up period, a total of 34 patients were enrolled in this study.

Signed informed consent was obtained from patients’ parents or their caregivers in accordance with the Code of Ethics of the World Medical Association (Declaration of Helsinki).

### Study Design

Patients enrolment to study was based on convenience in available, continuous and non-random manner.

Since the anticonvulsants could not be stopped due to ethical and therapeutic issues, a parallel control group was not considered and the patients’ clinical situation were compared with their own, before and after the intervention.

### Demographic and laboratory data

A questionnaire including demographic data and allergic signs and symptoms consisting of dermatological, upper and lower respiratory and gastrointestinal complaints was prepared and completed for each participant. All patients underwent diagnostic evaluations including complete blood count (for eosinophilia), serum specific IgE measurement and skin prick test for at least 6 common food allergens (cow’s milk, egg, peanut, soy, wheat and sea foods) and aeroallergens (house dust mite and common mixed aeroallergens).

A wheal of 3 mm or greater compared with negative control (saline) in skin prick test was considered positive.

Those with previous history of immediate reaction to food allergens, with development of objective or convincing symptoms after ingestion of the offending food and positive skin prick tests were diagnosed as IgE-mediated food allergy and were excluded from the study.

### Dietary Protocol

Since six common foods are implicated in triggering food allergy with IgE- and non IgE-mediated mechanisms and due to the lack of standard allergic tests in delayed type food allergies with non IgE-mediated mechanisms, a six food elimination diet remains as an appropriate diagnostic and therapeutic tool in patients with negative skin prick test^23^. Therefore, all patients with positive skin prick test and negative history of immediate reactions were considered as mixed type (IgE and non-IgE mediated) and those with negative skin prick test were regarded as non-IgE (cell-mediated) type food allergies. Dietary avoidance was recommended for both groups. Those children with refractory seizures who met the inclusion criteria and were compliant with the dietary protocol, were treated according to the following protocol.

For breastfed infants less than 6 months of age, a six-food elimination diet in addition to the specific allergens with positive results in skin prick test was initiated for their mothers and exclusive breast feeding for their nursing infant was implemented. In formula-fed infants less than 6 months of age, elemental based formula (Neocate, Nutricia) was prescribed exclusively. For breastfed infants over six months of age, a six-food elimination diet (cow’s milk, egg, peanut, soy, wheat, sea foods) in addition to the specific offender food with positive results in skin prick test were initiated for both mother and the nursing child. In formula-fed infants, a Neocate formula in addition to a six food elimination diet was started. In children, a six-food elimination diet (cow’s milk, egg, peanut, soy, wheat, sea foods) in addition to the specific offender food with positive results in skin prick test was started.

All the infants were continuously being monitored for any deviation from their growth and developmental curve parameters during a total period of twelve weeks (8 weeks of implementing the above mentioned dietary intervention and for the 4 following weeks after). In the case of any growth and developmental abnormalities, the patient was excluded from the study to avoid further developmental delay.

### Primary Outcome Measures

The primary outcome measures were defined as any reduction in seizure frequency after implementation of the hypoallergenic diet. The results of this study were evaluated in three distinct groups as complete elimination of seizures, or >90% reduction in seizures,or >50% reduction in seizures^15,16^.

#### Ethical Code

This research is confirmed in Iranian Registry of Clinical Trials with registration reference of **IRCT20171022036925N2** on **10/03/2018 (**www.irct.ir/user/trial/27457/view**)**.

The study protocol was approved by the Ethics Committee of Mofid Children Hospital. It is worth mentioning that all the information was confidential and all the appropriate measures have been taken to insure patients’ confidentiality.

### Statistical Analysis

This study used common statistics to summarize data and to describe the variables of a data set and descriptive statistics such as domain, mean and standard deviation.

After completing the forms, statistical analysis was performed using Paired t-Test, *X*^2^ and Fisher’s exact test. SPSS software version 16 was used. In all tests p < 0.05 was considered significant.

## Results

The youngest patient was 3 months old and the oldest was 16 years old with a mean age of 4 year (±3.5 SD). Seventeen patients (50%) were female and 17 were male (female/male ratio was 1). The mean age of seizure onset was 2 years (±2.5 SD). The mean age for initiation of anticonvulsant drugs was 3 years (±3.2 SD) (with a minimum age of one month and a maximum age of 10 years). A careful history revealed an allergic disorder in 29 of those patients (85.3%). Sixteen patients (47.1%) had cutaneous allergic involvement, 17 patients (50%) had some types of upper respiratory tract allergies and 21 patients (61.8%) had lower respiratory tract allergies. Gastrointestinal allergies were detected in 13 patients (38.2%). There were no complications including weight loss in patients receiving hypoallergenic diets in this study.

The patients’ data have been summarized in Table 1.

**Table 1:** A summary of patients’ records.

Cases	Age	Sex	Frequency of daily seizures Before food avoidance	Frequency of daily seizures after food avoidance
1	8 year	M	10	0
2	2 year	F	1	No response
3	2 year	F	2	No response
4	2.5 year	F	2	4 times a week
5	4 year	F	2	0
6	10 year	F	3	0
7	2.5 year	F	2	0
8	4 year	M	1	Once a week
9	10 year	M	3	0
10	9 month	F	2	Twice a week
11	2.5 year	f	1	Once a week
12	12 years	f	Once a week	0
13	5 year	F	5	Once a week
14	3 year	M	4	Once daily
15	9 year	M	5	Once daily
16	1.5 year	M	Once a week	0
17	4 month	M	2	Twice a week
18	2.5 year	F	3	0
19	2.5 year	M	5	0
20	1 year	f	10	0
21	6 year	f	2	0
22	6 month	M	5	Once a week
23	6.5 year	F	4 times a week	No response
24	5 year	M	3 times a month	0
25	13 years	M	3	0
26	6 year	F	4	0
27	4.5 year	M	3	Once a week
28	5 year	M	10	0
29	2.5 year	M	1	0
30	4 year	M	Once a week	0
31	14 month	F	4	No response
32	9 month	F	6	4 times a week
33	1 year	M	5	No response
34	11 years	M	10	Not cooperative

Table 2 shows EEG and MRI findings in the patients.

**Table 2:** Seizure types with MRI and EEG findings of the patients.

Type of seizures	Generalized	31(n)	91.1%
	Partial	3 (n)	8.8%
EEG findings	Normal	6 (n)	17.6%
	Abnormal	28 (n)	82.4%
MRI findings	Normal	22(n)	68%
	Abnormal	10(n)	31.3%

The most implicated food in skin prick tests were hen’s egg (32.4%), cow’s milk (20.6%), soy (20.6%) and peanut (20.6%), respectively (Table 3).

**Table 3:** Sensitivity to food allergens detected by skin prick test in patients.

Food Allergens	Positive Prick Tests N(%)	Negative Prick Tests N(%)
Cow’s milk protein	7 (20.6%)	27 (79.4%)
Hen’s egg	11 (32.4%)	23 (67.6%)
Fish	2 (5.9%)	32 (94.1%)
Wheat	4 (11.8%)	30 (88.2%)
Soy	7 (20.6%)	27 (79.4%)
Peanut	7 (20.6%)	27 (79.4%)
Tree nuts	2 (5.9%)	32 (94.1%)
All food allergens	25 (73.5%)	9 (26.5%)

Tables 4 and 5 demonstrate that house dust mite with 11.8% and tree pollens with 23.5% positivity in skin prick test were the most common indoor and outdoor aeroallergens in the studied patients, respectively.

**Table 4:** Sensitivity to indoor aeroallergens detected by skin prick test in patients.

Indoor Aeroallergens	Positive Prick test N(%)	Negative Prick Test N(%)
Aspergillus	0	34 (100%)
Alternaria	1 (2.9%)	33 (97.1%)
Mites	4 (11.8%)	30 (88.2%)
Cat	2 (5.9%)	32 (94.1%)
Dog	0	34 (100%)
Feather	0	34 (100%)
Cockroach	0	34 (100%)
Other indoor allergens	0	34 (100%)
All indoor allergens	6 (17.6%)	28 (82.4%)

**Table 5:** Sensitivity to outdoor aeroallergens detected by skin prick test in the patients.

Outdoor Aeroallergens	Positive Prick Test N(%)	Negative Prick Test N(%)
Grass	6 (17.6%)	28 (82.4%)
Weeds	6 (17.6%)	28 (82.4%)
Trees	8 (23.5%)	26 (76.5%)
Other outdoor allergens	0	34 (100%)
All outdoor allergens	12 (35.3%)	22 (64.7%)

Allergic Nasal and dermatological symptoms including nasal congestion and eczematous dermatitis were the most presented clinical sign and symptoms (Table 6).

**Table 6:** Clinical signs and symptoms of allergy in the patients.

Signs & Symptoms	Negative N(%)	Positive N(%)
Hives	1 (2.9%)	33 (97.1%)
Eczema	16 (47.1%)	18 (52.9%)
Itching	1 (2.9%)	33 (97.1%)
Other symptoms of skin allergy	16 (47.1%)	18 (52.9%)
Nasal congestion	16 (47.1%)	18 (52.9%)
Sneezing	9 (26.5%)	25 (73.5%)
Snoring	13 (38.2%)	21 (61.8%)
Other symptoms of upper respiratory tract allergy	17 (50%)	17 (50%)
Cough	5 (14.7%)	29 (85.3%)
Physician diagnosed asthma	4 (11.8%)	30 (88.2%)

Positive skin prick test was detected in 27 patients (79.4%). Twelve patients (35%) were sensitive to outdoor allergens and 6 (17.6%) had sensitivity to indoor allergens. The most common allergens among outdoor allergens were trees, grass and weed (23.5%, 17.6% and 17.6% respectively). Mites (11.8%) and cats (9.5%) were the most common indoor allergens detected by skin prick test. The definitive dominancy of food allergens is well demonstrated, as 73.5% of the positive skin prick tests were due to food allergens including egg (23.4%), cow’s milk (20.6%), soy (20.6%) and peanut (20.6%).

The daily average number of seizures in the studied population prior to intervention (food elimination diets) was 7.3 ± 0.4. It was reduced to 3.1 ± 0.5 after 8 weeks of strict diet. The mean rate of response to the intervention was 85.2% (±32.8 SD). Six patients (17.6%) demonstrated 50% to 89% reduction in the number of seizures, a significant decrease in seizures (90–99%) was also shown in other 6 cases (17.6%) and 17 patients (50%) became totally seizure free after 8 weeks of restrict dietary intake. These findings remained unchanged after 4 weeks of follow-up.

All of these findings were significant and meaningful. Only 5 patients did not show any significant response to therapy with no changes in their daily seizure frequency with elimination diets. There was no significant relationship between the response to therapy (defined as more than 50% reduction in the number of daily seizures after implementation of an 8-week period of elimination diets) and age (p = 0.49), gender (p = 0.33), skin prick test results (p = 0.1) and the type of seizure (p = 0.1).

## Discussion

The purpose of this study was to determine the relationship between refractory seizures and food allergies as an important cause of intestinal inflammation and consequently to investigate the role of food avoidance in certain patients with evidence of food allergy to control refractory seizures. Therefore, we sought for the effect of hypoallergenic diets on refractory seizures and we found a significant decline in the rate of daily seizures not only after an eight-week allergen elimination diet but also in the following 4 weeks of follow-up (p < 0.001).

There is a wealth of experience about the role of ketogenic diets on refractory seizures^18–27^.

The results on less than 20% seizure free after institution of ketogenic diet 24) compared to our results with hypoallergenic diets showing seizure freedom in 50% of patients and seizure reduction in more than 85% of patients during 12 weeks of elimination diet, are convincing.

It is known that allergic diseases are very common conditions with inflammation accounting for their main patho-mechanism^25–27^. In a study performed by Silverberg *et al*., epileptic children were found to have a high prevalence of allergic disease (~46%)^22,26,28,29^. Therefore, they suggested that epilepsy may be a comorbidity of severe childhood allergic disease^22^. Similarly, we noticed the following allergic symptoms including chronic dermatitis, lower respiratory tract allergy symptoms, and gastrointestinal tract symptoms including gastroesophageal reflux, chronic constipation, diarrhoea, abdominal cramp and perianal symptoms in the medical histories of twenty-nine patients (85%) in the present study.

The role of food allergens specially cow’s milk protein and wheat, as triggering factors in seizures have been emphasized by few case reports in literature. Egger studied the role of oligo-antigenic diets on 63 children with epilepsy whose seizures ceased during the diet therapy^30^. Lucarelli suggested the role of cow’s milk allergy in children suffering from Rolandic epilepsy with complete clinical remission in 85% of the cases with a cow’s milk free diet and reappearance of the seizures with reintroduction of cow’s milk into diet^31^. Kok *et al*. reported an infant presented with anaphylaxis to wheat who had developed generalized tonic clonic seizures 1.5 hours after initial reaction. Her skin prick test to wheat became positive later at 13 months old. The authors concluded that the complex immunological mechanism regulating the allergic reaction to food might play a significant role in this epileptic manifestation^28^.

In addition, concomitant seizure disorder has been reported in eosinophilic gastrointestinal disorder (EGID)^32^.

There are growing evidence emphasizing the role of inflammation as the main cause of seizure^8,29^.The proposed mechanisms underlying the peripheral inflammation causing seizure activity indicate the interaction between the nervous and the immune systems^14^ and implicate inflammation-related elements including cytokines, endogenous opioids, the nitric oxide signalling pathway and prostaglandins^33^. There are considerable reasons to believe that allergy may act as seizure precipitant in refractory epilepsies^31^.

Riazi *et al*. suggested that systemic inflammation may induce a mirror inflammatory response in the brain with short or long-term effects on seizure susceptibility^8^. Any disruption in blood brain barrier due to peripheral inflammation may play a key role in this regard^14^. A definitive role is suggested for pro-inflammatory cytokines including IL-1β and TNF-α in the brain and particularly in neuronal transmission^34,35^. In Silverberg study on mice a single tonic-clonic seizure induced IL-4 and IgE-negative T and B cells to infiltrate brain; therefore, suggested that inflammatory pathways in allergic diseases may be activated in brain which contribute toward epilepsy^22^. Riazi *et al*. also identified a microglial-dependent increase in CNS excitability mediated by TNF which is due to a potential link between peripheral and central inflammation^36^. Falsaperla and Vitaliti presented 3 cases with gastroenteritis involvement as the site of peripheral inflammation. They suggested a peripheral inflammation due to food allergy involving the gastrointestinal tract with subsequently stimulated antigen presenting cells, T helper2 lymphocyte subsets and their secretion of pro-inflammatory cytokines. Durkin *et al*. suggested milk proteins in cow’s milk allergic patients as the major cause for gastrointestinal inflammation, and hence, induction and migration of T-lymphocytes sensitized to cow’s milk protein within the blood brain barrier^13^. Epilepsy also has been reported as a documented neurological manifestation of celiac disease with a prevalence of 0.8–6%^37^. Gluten sensitivity may also present with neurological manifestations so that the underlying disease may easily become unrecognized and untreated^37^.

As gastrointestinal tract is a prominent organ involved in non-IgE mediated food allergy, it can be assumed that allergic inflammation in gastrointestinal tract (as a peripheral cause of inflammation) could be the reason for the seizures in our patients. A recently raised theory called gut brain axis, suggests a bidirectional communication between gut and brain which may be a justification for our claim^38,39^.

Our findings may suggest the possibility of a better seizure control with least pharmacological intervention; however, lack of a parallel simultaneous control group might be considered as a limitation to this study. Other limitations include non-randomized patient selection, lack of a control group without any treatments, small sample size, short duration of the study and possibility of patients’ poor compliance with the dietary regime. With regard to the high effect size in our study, the effect of confounders could be ignored. A blinded study on food eliminations is needed to confirm the outcome of this dietary study and further studies may characterize the specific role of cytokines in the development of refractory epilepsies in food allergy.
